# Implementation outcomes of a symptom management intervention in ambulatory oncology practices evaluated using a cluster randomized stepped-wedge trial design

**DOI:** 10.1186/s13012-025-01475-y

**Published:** 2025-12-02

**Authors:** Justin D. Smith, Katy Bedjeti, Nicola Lancki, Elizabeth A. Sloss, James L. Merle, Sheetal Kircher, Ava Coughlin, Susan Metzger, Kimberly A. Webster, Mary O’Connor, September Cahue, Ann Marie Flores, Quan Mai, Betina Yanez, Michael Bass, Roxanne E. Jensen, Ashley Wilder Smith, Allison J. Carroll, Cynthia Barnard, Christopher M. George, Dean G. Tsarwhas, Kimberly Richardson, Frank J. Penedo, Karla Hemming, Sofia F. Garcia, Denise M. Scholtens, David Cella

**Affiliations:** 1 Department of Population Health Science, Division of Health System Innovation and Research, Spencer Fox Eccles School of Medicine at the University of Utah, Salt Lake City, UT USA; 2 Department of Medical Social Sciences, Northwestern University Feinberg School of Medicine, Chicago, IL USA; 3 Department of Preventive Medicine, Northwestern University Feinberg School of Medicine, Chicago, IL USA; 4 College of Nursing, University of Utah, Salt Lake City, UT USA; 5 Northwestern Medicine, Chicago, IL USA; 6 Department of Physical Therapy and Human Movement Sciences, Northwestern University Feinberg School of Medicine, Chicago, IL USA; 7 Information Technology, Northwestern University Feinberg School of Medicine, Chicago, IL USA; 8 Outcomes Research Branch, Healthcare Delivery Research Program, National Cancer Institute, Rockville, MD USA; 9 Department of Psychiatry and Behavioral Sciences, Northwestern University Feinberg School of Medicine, Chicago, IL USA; 10 Black Cancer Collaborative, Chicago, IL USA; 11 Departments of Psychology and Medicine, University of Miami, Miami, FL USA; 12 Institute of Applied Health Research, University of Birmingham, Birmingham, UK

**Keywords:** Cancer symptom management, Electronic patient-reported outcomes, Hybrid effectiveness-implementation study, Multilevel implementation strategy, Patient adoption, Stepped-wedge

## Abstract

**Objective:**

To test a package of clinician- and system-level implementation strategies on the adoption and reach of an electronic health record (EHR)-integrated cancer symptom assessment and management program, called cPRO, within a large academic healthcare system.

**Methods:**

This hybrid type 2 effectiveness-implementation study used a cluster randomized stepped-wedge trial design to test a package of strategies targeting system operations, clinician practices, and patient experience to support implementation of cPRO. Six clusters, comprised by 26 oncology clinic sites, were randomly allocated to one of six sequences which dictated the time at which each cluster underwent a 6-month implementation preparation period followed by a transition to the post-implementation phase in which 46 discrete implementation strategies were deployed. The primary implementation outcome was patient adoption of cPRO, measured by the proportion of patients completing cPRO assessments. Secondary outcomes included the reach of patient enrollment in the cPRO system and clinician adoption of referrals using an EHR “dot phrase” (snippets of text that can be quickly inserted into patient charts for referrals, orders, etc.) triggered by elevated cPRO scores. Data were analyzed using a cluster-period level analysis (generalized least squares linear regression with fixed cluster effects and adjustment for calendar time).

**Results:**

The study included 34,643 unique outpatients receiving cancer treatment at 26 clinics between October 2020 and March 2024. The primary analysis showed no significant difference between the pre- and post-implementation periods on the mean difference in the proportion of patients who complete the assessments (25% vs. 40%). Secondary outcomes indicated that the implementation strategy package did not significantly improve the reach of cPRO enrollment among patients (RR = 1.00, CI: 0.78 to 1.27). Clinician adoption of referrals in response to elevated cPRO symptom scores showed a marginal positive, alebeit non-statistically significant association with the implementation strategy package (RR = 1.66, CI: 0.79 to 3.48), although this varied over time.

**Conclusions:**

The implementation strategies tested did not significantly alter patient adoption rates of cPRO when comparing pre- and post-implementation periods, but might improve clinician adoption of the EHR dot phrase function. Future studies should explore strategies to enhance the integration of digital symptom management systems into routine cancer care to improve patient outcomes.

**Trial registration:**

ClinicalTrials.gov NCT03988543; registered 8 May 2019 https://clinicaltrials.gov/study/NCT03988543?term=NCT03988543&rank=1.

**Supplementary Information:**

The online version contains supplementary material available at 10.1186/s13012-025-01475-y.

Contributions to the Literature

- Despite null findings on patient adoption and reach of cPRO, the implementation strategies successfully improved clinician adoption of an EHR function (i.e., dot phrase) to automatically refer patients with elevated symptom scores to appropriate management services.
- Findings highlight the difficulty of improving adoption and reach of cancer symptom monitoring interventions in a large health system, despite using a package of evidence-informed implementation strategies targeting multiple system levels and individuals.
- This study illustrated some of the challenges of cluster randomized stepped-wedge designs in implementation research, including implementation strategy exposure timing when strategies cannot be turned on precisely at prespecified crossover points, which was further complicated due to the COVID-19 pandemic.

## Background

Individuals diagnosed with cancer experience symptoms related to disease processes and treatment that negatively impact quality of life, even after treatment completion [1–3]. Electronic patient-reported outcome (PRO) systems to monitor and intervene for on symptoms have demonstrated efficacy in reducing symptom burden and improving health outcomes, such as lower unplanned healthcare utilization and improved survival [4–7]. While implementation of these systems is feasible, integration into routine cancer care remains limited [8, 9].

Well-documented barriers to implementation of electronic PRO systems range from individual characteristics to healthcare system factors [10, 11]. Patient- and clinician-level barriers include low engagement, hesitancy to use the systems, and increased clinician workload or time burden. System-wide barriers include lack of integration with the electronic health record (EHR), lack of reimbursement, and fragmented care transitions caused by limited downstream referral workflows and resources for symptom management [12, 13]. Inconsistent implementation of digital symptom management tools, like electronic PROs, can exacerbate health disparities, particularly among vulnerable populations [13].

Previous studies of the implementation of electronic PRO systems in cancer care highlight various implementation strategies and outcomes [14, 15]. Recommended implementation strategies include promoting engagement (e.g., education/training, alerts/reminders), appropriate workflow design, and integration with existing EHR systems [12, 16, 17]. Deploying, evaluating, adapting, and activating strategies for electronic PRO implementation have also been described [14]. Yet, evidence related to the effectiveness of real-world implementation of electronic PRO symptom management systems into routine cancer care remains limited. Further, evaluation of pragmatic implementation strategies to optimize electronic PRO symptom monitoring across large academic medical centers remains under-explored.

### Northwestern University IMPACT (NU IMPACT) study

The Northwestern University IMPACT (NU IMPACT) study is one of three trials that comprise the Improving the Management of symPtoms during And following Cancer Treatment (IMPACT) Consortium [18], funded by the National Cancer Institute. The IMPACT Consortium, a cooperative agreement comprised of three research centers [19–21] and a coordinating center, aims to improve symptom control for patients during cancer treatment and survivorship by building the evidence base of successful strategies for implementing and scaling effective cancer symptom management into routine clinical care. The NU IMPACT study is a hybrid type 2 effectiveness-implementation study comprised of separate randomized trials: (1) a cluster randomized stepped wedge trial testing a package of *implementation* strategies on the adoption and reach of an EHR-integrated cancer symptom assessment and management program (called cPRO)—reported here; and an individually-randomized trial testing the clinical *effectiveness* of cPRO plus a web-based patient-tailored symptom self-management toolset (which we referred to as enhanced care in the effectiveness RCT; consisting of patients with elevated symptoms being offered the opportunity to create an account in a custom website and smartphone app that provides tailored education, symptom management strategies, and guidance) compared to usual care (cPRO) in which symptoms were managed solely by the care team [19].

The clinical effectiveness trial results are reported separately [22]. In brief, among 1,614 (*n* = 804 in enhanced care, *n* = 810 in usual care) adult cancer patients and survivors enrolled in the patient-level randomized controlled trial (RCT), PROMIS measures of anxiety, depression, fatigue, pain interference, and physical function collected at baseline and monthly for 12 months did not show any statistically significant differences between groups on symptom burden.

In this part of the trial, we implemented and evaluated the effect of an implementation strategy package to promote the adoption and reach of cPRO across 26 ambulatory oncology clinics in a large academic health center [23, 24]. cPRO plus (enhanced care version) was not part of this implementation test as it was delivered through a separate RCT. Here we report results of the cluster randomized stepped-wedge trial on primary and secondary *implementation* outcomes. Our overarching implementation research question was: What is the impact of a multicomponent, multilevel implementation strategy package on both patient and clinician adoption and reach of cPRO? Specifically, we examined how the package of implementation strategies affected patient adoption of cPRO, reach of enrollment in the patient portal, and clinician adoption of automated referrals when elevated cPRO scores triggered an alert.

## Methods/design

### Site/setting

In total, there were potentially 7 eligible clusters that included 30 ambulatory oncology clinic sites across the Northwestern Medicine (NM) seven-hospital healthcare delivery system. NM serves the nine-county Chicago metropolitan area with a population of more than 8.6 million people. The NM cancer care network includes the Robert H. Lurie Comprehensive Cancer Center in downtown Chicago, plus 11 community-based cancer centers across the Chicago metropolitan area. Study clusters were determined by existing administrative and organizational units within the healthcare system and ranged from 1 to 9 clinics (a study cluster is defined as a natural grouping of cancer clinics). Clinics within each cluster follow naturally occurring administrative structures within the health system (e.g., shared operational leadership). Clinics focused on medical oncology, hematology/oncology, gynecological oncology, and surgical oncology. Of the 7 potential clusters, 6 clusters (26 clinics) were included in this randomized evaluation. The other remaining cluster (of *n* = 4 clinics) was pre-specified by the health system based on readiness to start first in the roll-out before randomization and thus is not included in the primary analysis).

### Study design

**Fig. 1:**
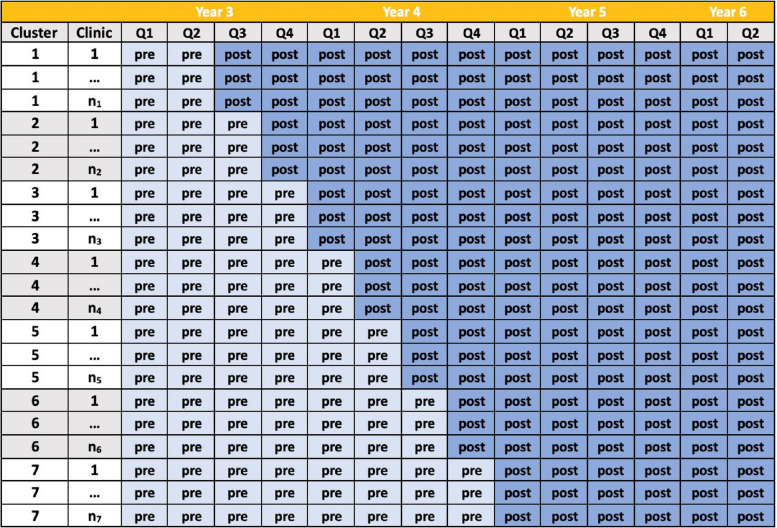
Cluster Randomized Stepped-Wedge Trial Design Note. Cluster 1 was removed from the dataset for all analyses as it was not randomly assigned

We conducted a hybrid type 2 effectiveness-implementation study [25] using a cluster randomized stepped-wedge trial design to test the impact of a multicomponent, multi-level package of implementation strategies on implementation outcomes. Sample size justification can be found in the protocol papers [19, 26]. The 6 clusters were randomly allocated to one of 6 sequences which dictated the time at which the cluster underwent a 6-month implementation preparation period in which the implementation strategies were initiated to begin just prior to or at the point of each cluster’s transition into implementation, called the ‘post-implementation’ phase. Times of transition from pre- to post-implementation occurred every three months over 3.5 years. This 3-month step was chosen for logistical reasons such that the design would fit within the 5-year project period and allow for allocation of implementation support resources to be sufficiently spread out. The study design and analytic plan afforded 42 monthly proportions from 26 clinics in the 6 randomized clusters, for a total of 1092 monthly proportions to be used for analysis. The total duration of the stepped-wedge trial was 42 months (October 2020—March 2024), with a range of time after exposure to the implementation strategy package spanning 33 (first randomized cluster) to 18 months (last cluster). Because of our prior experience and preliminary work in the NM system, a sensitivity analysis (described in the Analysis section) was chosen based on considering that some strategies in the package might be in place slightly before or after the official crossover. The implementation roll-out was originally scheduled to begin in March 2020, the month when healthcare systems around the country were required to shift their care model dramatically due to the onset of the COVID-19 pandemic. Ethics approval for this study was obtained from the Northwestern University Institutional Review Board with a waiver of informed consent. Figure 1 presents a diagram of the cluster randomized stepped-wedge design [27].

#### Inclusion criteria (patients)

The inclusion criteria are outpatients who were receiving either curative or non-curative cancer care, or in post-treatment survivorship and met the following criteria: age ≥ 18 years; cancer diagnosis in medical record (ICD-9/ICD-10 code on problem list or encounter diagnosis); and had a clinic visit during the study period and were sent a cPRO invite. Due to differences between participants enrolled in and consented to the effectiveness RCT (above) that might bias implementation outcomes (e.g., consent to participate and engage with the cPRO system, more frequent cPRO assessments to track symptoms over time, participant incentives for completion and participation), data from patients in the RCT were not included in the implementation sample.

#### Inclusion criteria (clinicians)

All oncology clinicians practicing in enrolled clinics in the six clusters were included to assess implementation outcomes such as reach, adoption, clinician follow-up to alerts, and clinical referrals to appropriate services. Across the 26 clinics included in the analysis, 170 clinicians (e.g., Medical Doctor, Advanced Practice Registered Nurse, Social Worker) contributed data.

#### cPRO (the usual care condition in the trial)

The cPRO program offers tailored resources to the patient as relevant to symptom burden. The patient is offered the opportunity to complete a symptom assessment in English or Spanish through the patient portal, if they have an account; or they will receive a telephone outreach, or an in-clinic offer to complete the assessment. cPRO assesses key patient-reported symptoms (depression, anxiety, fatigue, pain interference, and physical function) using Patient Reported Outcomes Measurement Information System® (PROMIS®) measures [24] along with two supportive oncology care checklist items and is available in both English and Spanish. Elevated symptom scores and endorsed supportive care needs trigger clinician alerts via EHR in-basket messaging to enhance clinical decision-making. If the assessment reveals symptom burden, the clinician is advised by EHR inbox message and can acknowledge the results with a predefined EHR one-click function (i.e., dot phrase), which will generate a referral for support.

Prior to this trial and during the pre-implementation phase, consistent with the 2020 Standards of the American College of Surgeons’ Commission on Cancer (CoC), routine symptom monitoring was in place. However, rates of uptake were ~ 25%. At the start of this trial, NM’s office of medical information technology made the cPRO symptom assessments available all clinics in the system. However, clinics were not provided with information or any other form of implementation support for cPRO until they crossed over in the rollout sequence to post-implementation, at which point, the implementation strategy package began and cPRO became the primary symptom monitoring tool as opposed to other options that had been used previously (e.g., the National Comprehensive Center Network’s Distress Thermometer [27]). Thus, while cPRO was available in Epic during the pre-implementation phase, implementation strategies were only deployed in accordance with the clinic’s position in the stepped wedge trial’s rollout schedule.

#### Implementation strategies (the intervention condition in the trial)

A total of 46 discrete implementation strategies were used. The study team classified strategies into three categories: 1) those that focus on system and clinical team processes to support implementation, 2) those that target clinicians and their capacity, capability, and motivation to deliver the intervention, and 3) those that target patient engagement and involvement in the implementation process.

##### System and clinical team strategies

Two-thirds of the strategies used (*n* = 31/46, 67.4%) were aimed at system and team processes. These strategies were broadly used to improve the reach of cPRO alert responses and referrals by the clinical team, with secondary targets of acceptability, feasibility, and sustainment of cPRO. Examples of strategies in this category included: building a coalition by cultivating relationships between quality improvement leaders and healthcare system leaders to establish buy-in and determine staffing and work infrastructure logistics; setting a NM-wide committee to guide cPRO implementation across specialties; identifying early adopters and practice champions and developing educational materials to foster their involvement in supporting the implementation process; designating and recruiting administrators for leadership; updating the health information technology infrastructure to facilitate implementation of cPRO on tablet computers and develop a template for quality assurance processes; and developing and sharing regional implementation blueprints to guide workflows, data collection, and execution of delivery.

##### Clinician strategies

Almost one-quarter of the strategies directly targeted clinicians (*n* = 11/46, 23.9%) and used to improve adoption of cPRO alerts and referrals from cPRO triggers (secondarily targeting acceptability and sustainability). Examples of strategies in this category included: developing educational materials, such as a training manual, a clinician-facing video, and a toolkit to make it easier for clinicians to learn about and deliver cPRO; using train-the-trainer techniques; providing centralized technical assistance to support clinicians during active implementation of cPRO and problem-solve workflow issues; and facilitating the relay of cPRO results and alerts to clinicians via the EHR to support decision-making.

##### Patient strategies

A few strategies (*n* = 4/46, 8.7%) targeted patients to increase the adoption of and engagement with cPRO by providing access to knowledge and information about cPRO and improving capability and opportunity to be active participants in their care. Patients received educational materials and automated emails, calls, or text messages directing them to access the patient portal; the research and healthcare system collaborated to develop posters, pamphlets, and fliers in both English and Spanish and distributed them to clinics.

### Outcome measures

As specified in the updated statistical analysis plan for the NU IMPACT study [26] that was published prior to data analysis, the primary implementation outcome is patient adoption of cPRO, as measured by clinic-level monthly proportions of patient engagement with the EHR-based cancer symptom monitoring system. Our operational definition of patient adoption was operationalized as the proportion of patients who completed a cPRO assessment among those eligible. This is consistent with the Reach, Effectiveness, Adoption, Implementation, and Maintenance (RE-AIM) evaluation framework [28] with guidance from a paper on implementation outcomes for digital intervention technologies [29]. Secondary implementation outcomes were: 1) reach of cPRO, defined as the proportion of patient visits where the patient was offered cPRO, either through their patient portal account, telephone outreach, or in-clinic assessment among those eligible for cPRO, 2) proportion of patients who are referred for appropriate services (as measured by dotphrase usage) from among those that trigger an alert in cPRO and 3) clinician adoption, defined as the proportion of unique clinicians who ever used the cPRO EHR function (e.g., dot phrase) to follow up on an alert received from patients (numerator) among any unique clinicians whose patients triggered an alert during the time period of interest (denominator).

Use of and adaptations to implementation strategies were captured using the Longitudinal Implementation Strategies Tracking System (LISTS) [30], a method developed by members of the IMPACT Consortium’s Implementation Science Working Group that uses a timeline follow-back procedure to limit retrospection bias when reporting on strategy use and adaptations across the study period. Members of the NU IMPACT research team, which included investigators, clinician champions, operations leaders, and project coordinators who were knowledgeable about strategies being used for the study, met at at minimum every three months for 1–2 h meetings beginning in June, 2020 until August, 2024. The LISTS method incorporates strategy reporting and specification guidelines from Proctor, Powell, and McMillen [31] with elements of the Framework for Reporting Adaptations and Modifications Expanded to Evidence-based Implementation Strategies [32] (e.g., the When, Who, how Widespread, Planned/Unplanned data elements). Given the extensive data collected via the LISTS method, a detailed report of strategies used and their adaptations during the trial will be published separately.

### Data collection

Data for reach and adoption metrics were pulled from NM’s Enterprise Data Warehouse at the end of the study period. Additional measures concerning acceptability, appropriateness, and perceived sustainability were assessed using surveys administered electronically via REDCap (Research Electronic Data Capture) [32] 12 months after implementation began for each cluster in the stepped-wedge roll-out sequence. These results will be reported elsewhere in a secondary implementation outcomes paper.

### Statistical analysis

A statistical analysis plan for implementation outcomes was developed and published prior to completion of follow-up and prior to statistical analysis of the data [26]. Briefly, differences in the primary implementation outcome for post- versus pre-implementation were estimated using generalized least squares linear regression with a lagged autoregressive error structure and adjustment for cluster and time effects to model underlying secular trends [33]. We performed a sensitivity analysis in lieu of an a priori transition period, which excluded the data from each month (two months total) on either side of the crossover point. Exploratory exposure time indicator (ETI) models were used to model a gradual, rather than instantaneous, impact of the implementation strategies on study outcomes using a categorical variable for time (quarter) since implementation and time average treatment effects were estimated from these models [34]. Similar approaches were used for evaluation of secondary implementation outcomes of reach of patient enrollment in cPRO and clinician adoption of cPRO dot phrases to address alerts for elevated cPRO scores.

## Results

### Descriptives

**Fig. 2:**
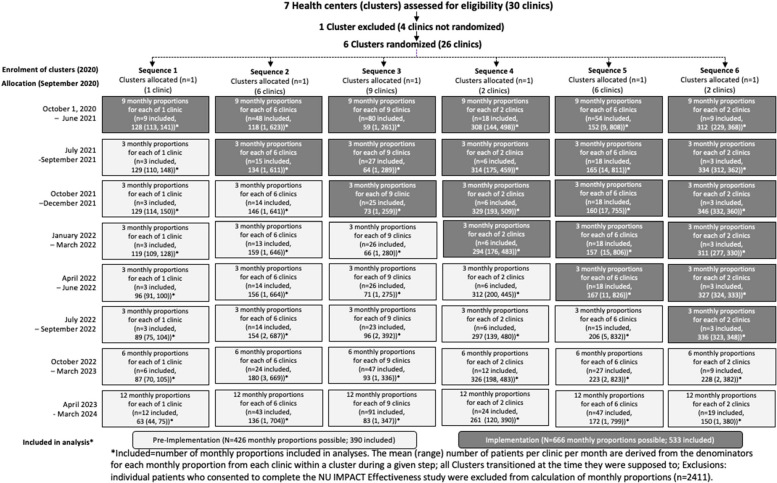
CONSORT Extension for Cluster-Randomized Stepped-Wedge Trial

Figure 2 presents the Consolidated Standards of Reporting Trials (CONSORT) extension for the stepped-wedge cluster randomized trial [35] (checklist provided as Additional File 1). The included sample consisted of 25,444 patients and 131,141 eligible visits for evaluation of adoption and 189,463 eligible clinic visits for evaluation of reach. The number of unique patients by cluster over the study period ranged from 1221 to 7397, with a mean of 4492 (*SD* = 2417). The number of cPRO invites by clinic site ranged from 4122 to 37,077, with a mean of 21,857 (*SD* = 11,638). The number of clinicians at each clinic in the analysis ranged from 11 to 53 (M = 33, SD = 13) in each cluster.

**Fig. 3:**
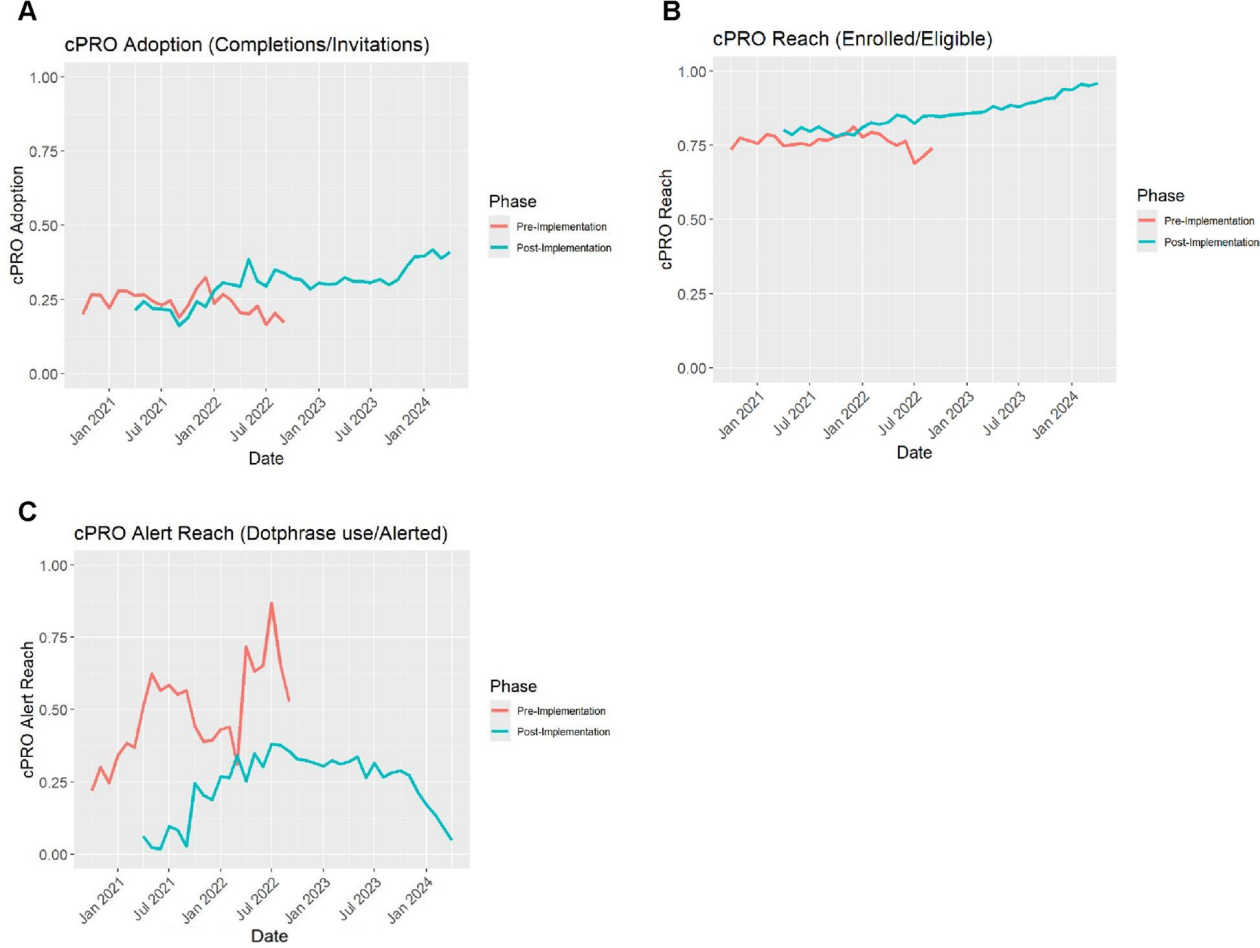
Implementation Outcomes by Pre- and Post-Implementation Phase Across Clusters

The primary outcome (patient adoption rate, defined as completing a cPRO assessment/total patient *n*) had a mean monthly adoption rate by cluster ranging from 0.23 to 0.43 across the study period (Table 1). In the pre-implementation phase, the range was 0.18 to 0.33, and in the post-implementation phase, the range was from 0.24 to 0.50. Overall, the rate of patient adoption of cPRO increased from 25% at the beginning of the study to 40% at the end of the study across the six included clusters (see Panel A in Fig. 3). An omnibus test of monthly adoption variation by cluster not accounting for pre-post change or time trends was significant (*F* = 29.23, *p* < 0.001), and linear model regressing monthly clinic-level adoption ratio on cluster showed that two clusters (mean differences 0.20, 95%CI 0.14 to 0.26, 0.09 95% CI 0.02 to 0.16, had higher overall adoption rates than the reference cluster. Table 1 shows the means and standard deviations of patient adoption and reach for each cluster by pre- and post-implementation periods and overall.

**Table 1:** Patient cPRO Adoption and Reach Rates by Cluster

	Sample size (Adoption and Reach denominator)			Adoption rate for cPRO (primary outcome)			Reach rate for cPRO enrollment (secondary outcome)		
	Pre	Post	Overall (both periods)	Pre	Post	Overall (both periods)	Pre	Post	Overall (both periods)
Cluster	n	n	n	Mean (sd)	Mean (sd)	Mean (sd)	Mean (sd)	Mean (sd)	Mean (sd)
1*	7925	49,188	57,113	0.24 (0.06)	0.26 (0.05)	0.25 (0.05)	0.78 (0.06)	0.86 (0.05)	0.85 (0.06)
2	1156	3022	4178	0.21 (0.04)	0.24 (0.06)	0.23 (0.06)	0.64 (0.04)	0.76 (0.09)	0.74 (0.1)
3	7650	18,639	26,289	0.23 (0.19)	0.31 (0.19)	0.28 (0.18)	0.74 (0.18)	0.88 (0.1)	0.83 (0.15)
4	8253	18,278	26,531	0.33 (0.17)	0.5 (0.27)	0.43 (0.25)	0.76 (0.24)	0.86 (0.19)	0.82 (0.21)
5	11,156	14,361	25,517	0.31 (0.04)	0.33 (0.04)	0.32 (0.04)	0.88 (0.03)	0.92 (0.03)	0.9 (0.04)
6	19,873	17,296	37,169	0.25 (0.1)	0.3 (0.15)	0.27 (0.13)	0.78 (0.12)	0.86 (0.09)	0.81 (0.11)
7	7769	4897	12,666	0.18 (0.05)	0.3 (0.28)	0.24 (0.21)	0.68 (0.03)	0.86 (0.13)	0.77 (0.13)

^*^Cluster 1 was removed from the dataset for all analyses as it was not randomly assigned

For the secondary outcome of patient reach rate of enrollment in the patient portal (enrolled/total patient *n*) in the analysis sample, the mean monthly clinic reach rate by cluster ranged from 0.74 to 0.90. In the pre-implementation phase the rate ranged from 0.64 to 0.88. In post-implementation, the reach rate ranged from 0.76 to 0.92. All sites increased their reach rate from pre- to post-implementation (see Panel B in Fig. 3). An omnibus test of monthly clinic reach variation by cluster not accounting for period or time trends was significant (*F* = 7.57, *p* < 0.001), and a subsequent linear model showed all clusters except one had significantly higher overall reach rates when compared to the reference cluster: meandifference (MD) 0.10 95% CI 0.04 to 0.15; MD 0.08 95% CI 0.03 to 0.14; MD 0.16, 95% CI 0.10 to 0.23; MD 0.07 95% CI 0.02 to 0.13; MD 0.04 95% CI −0.03 to 0.10. Table 1 shows the reach rates by cluster for cPRO enrollment for the pre- and post-implementation periods and overall.

For the secondary outcome of alert reach defined as proportion of alerting cPRO scores with dotphrase response, mean monthly pre-implementation alert reach was 47% and ranged from 4 to 70% across clusters. Post-implmentation mean of monthly alert reach was 27% ranging from 0 to 46% across clusters. There was an overall decline in alert reach from pre to post-implementation and within sites the rate of alert reach either remained the same (2%, 0.3%) or declined (pre to post: 1% to 0%; 51% to 46%; 59% to 35%; 57% to 38%; 70% to 44%) from pre to post. Usage varied over time with a peak in July 2022, as can be seen in Fig. 3 panel C. Notably, this month was the peak for both those in pre- and post-implementation clusters.

For the secondary outcome of clinician adoption of the cPRO dot phrase for referrals (clinicians ever used the dot phrase/total number of clinicians in dataset), across the entire study period 52% of clinicians used a cPRO dot phrase at least once. Clinician adoption of the cPRO dot phrase by cluster ranged from 3% of clinicians to 82% of clinicians (Cluster values: 3%, 61%, 79%, 11%, 79%, 82%). In a logistic regression model of clinician dot phrase adoption (dependent) and cluster (independent), with the exception of one cluster OR 4.00, 95% CI 0.55 to 80.60), all clusters had significantly higher rates of clinician adoption compared to the reference cluster OR 48.7, 95% CI 8.87 to 916; OR 115, 95% CI 19.6 to 2239; OR 118, 95% CI 21.70 to 2225; OR 140, 95% CI 16.3 to 3455).

Although clinic-level adoption was not a prespecified implementation outcome, it is worth noting that 32 clinics within the system were approached to participate, but two declined to participate (reason(s) unknown) resulting in a clinic-level adoption rate of 30/32 (94%).

### Primary outcome: patient adoption of cPRO

Patient adoption rates showed a general increase over time but the risk ratio (RR = 1.00, CI: 0.63–1.57) indicated no significant difference in adoption of cPRO between pre- and post-implementation periods. Effects for calendar time revealed significant increases in adoption in study quarters 13 and 14 compared to the initial quarter (Q13-Q1 mean difference: 0.21, CI: 0.12 to 0.29; Q14-Q1 mean difference: 0.20, CI: 0.11 to 0.29). Complete results of the primary and secondary implementation outcomes are in Table 2.

**Table 2:** Main Outcome Models

	**Adoption (primary)**			**cPRO Reach**			**Alert Reach**		
	**RR**	**95% CI**	***p*-value**	**RR**	**95% CI**	***p*-value**	**RR**	**95% CI**	***p*-value**
Post-Implementation (pre-implementation is reference)	1.00	0.63, 1.57	0.35	1.00	0.78, 1.27	0.44	1.66	0.79 to 3.48	0.10
	**MD**	**95% CI**	***p*-value**	**MD**	**95% CI**	***p*-value**	**MD**	**95% CI**	***p*-value**
Post-Implementation (pre-implementation is reference)	0.03	−0.03, 0.08	0.35	0.01	−0.02, 0.04	0.44	0.08	−0.01, 0.18	0.10
Time (categorical, Quarter 1 is reference)									
Quarter 2	0.01	−0.05, 0.06	0.86	0.00	−0.03, 0.04	0.80	0.21	0.10, 0.31	< 0.01
Quarter 3	0.01	−0.05, 0.06	0.84	0.03	−0.01, 0.06	0.13	0.18	0.08, 0.29	< 0.01
Quarter 4	−0.05	−0.11, 0.01	0.11	0.03	0.00, 0.06	0.06	0.13	0.02, 0.23	0.02
Quarter 5	0.03	−0.03, 0.10	0.27	0.04	0.01, 0.07	0.02	−0.02	−0.13, 0.09	0.72
Quarter 6	0.05	−0.02, 0.12	0.14	0.06	0.02, 0.10	< 0.01	−0.09	−0.21, 0.03	0.15
Quarter 7	0.06	−0.01, 0.13	0.08	0.07	0.03, 0.11	< 0.01	−0.06	−0.19, 0.06	0.32
Quarter 8	0.07	−0.01, 0.15	0.07	0.07	0.02, 0.11	< 0.01	−0.13	−0.27, 0.01	0.07
Quarter 9	0.04	−0.04, 0.12	0.33	0.08	0.04, 0.12	< 0.01	−0.19	−0.34, −0.05	0.01
Quarter 10	0.00	−0.08, 0.08	0.98	0.08	0.04, 0.13	< 0.01	−0.16	−0.31, −0.02	0.03
Quarter 11	0.04	−0.04, 0.12	0.33	0.11	0.06, 0.15	< 0.01	−0.20	−0.34, −0.05	0.01
Quarter 12	0.05	−0.03, 0.14	0.19	0.12	0.07, 0.16	< 0.01	−0.15	−0.30, −0.01	0.04
Quarter 13	0.21	0.12, 0.29	< 0.01	0.16	0.11, 0.20	< 0.01	−0.21	−0.36, −0.07	< 0.01
Quarter 14	0.20	0.11, 0.29	< 0.01	0.15	0.10, 0.20	< 0.01	−0.25	−0.41, −0.10	< 0.01
Clinic size (in 100 s of patients)	0.00	0.00, 0.00	0.40	0.00	0.00, 0.00	< 0.01	0.02	0.02, 0.02	< 0.01
Cluster (categorical, Cluster 2 is reference)									
Cluster 3	0.06	0.00, 0.12	0.06	0.12	0.09, 0.15	< 0.01	0.42	0.32, 0.52	< 0.01
Cluster 4	0.20	0.15, 0.26	< 0.01	0.13	0.10, 0.15	< 0.01	0.45	0.36, 0.55	< 0.01
Cluster 5	0.10	0.03, 0.17	< 0.01	0.19	0.15, 0.22	< 0.01	−0.12	−0.23, −0.01	< 0.01
Cluster 6	0.06	0.00, 0.12	0.05	0.09	0.06, 0.12	< 0.01	0.45	0.35, 0.55	< 0.01
Cluster 7	0.02	−0.06, 0.10	0.60	0.02	−0.01, 0.06	0.23	0.52	0.39, 0.65	< 0.01

CI = Confidence Interval. MD = Mean difference in the proportion of adoption and reach. Cluster 1 not included in these models

Sensitivity analyses did not meaningfully alter any of the primary outcome analysis results. Weighted sensitivity analysis in the generalized least squares model based on the number of unique eligible patients per clinic were used to evaluate the impact on the average individual. These models showed that in the fully adjusted model adoption decreased by around 10.5% in the post-implementation phase compared to the pre-implementation phase, and this effect was statistically significant (mean difference: −0.10, CI: –0.20 to –0.01). Further, in the weighted model, there was a small, significant negative effect of the clinic size on adoption (mean difference: −0.01 per 100 patients CI: −0.02 to 0.00). This suggests that patients at larger clinics had a slightly lower probability of seeing a provider that had adopted cPRO. A linear mixed effects model specifying a random effect for cluster, a random cluster by time effect, a random clinic effect, fixed effects for implementation, study quarter, clinic size, and a Kenward-Roger small sample correction was evaluated as a sensitivity analysis. An *F*-test using Kenward-Rogers correction comparing linear mixed effects models with and without the post-implementation variable showed no significant effect of post-implementation on adoption (*p* = 0.819), confirming that implementation status does not meaningfully alter adoption rates.

In an exploratory ETI analysis, coefficients for each level of exposure quarter, relative to the reference level (pre-implementation), as well as the overall time averaged treatment effect, showed no statistical significance (*p*-values > 0.05), indicating no strong evidence that the length of exposure to the implementation strategies significantly affected adoption rates.

### Secondary outcomes

Results comparing pre- to post-implementation phases, did not show a statistically significant effect on reach of cPRO in most models (e.g., risk ratios close to 1, *p*-values consistently > 0.05). Sensitivity analyses, including the transition period exclusion and the weighted model, confirmed this result. Although a slight effect was noted in some sensitivity models, the overall finding is that the implementation strategy package, in the presence of other covariates, did not meaningfully impact reach of cPRO. Time effects varied by quarter, with reach significantly increasing in study quarters 5, 6, and 8–14 compared to the reference quarter. This pattern was consistent across the initial model and the sensitivity analyses (with exception of the weighted model), suggesting a sustained upward trend in reach as the study progressed. Concerning cluster effects, four of the clusters consistently showed higher reach compared to the reference cluster in most models, with *p*-values < 0.05 in nearly all cases. Clinic size, measured by the number of unique patients, generally did not have a significant effect on reach across models. The weighted model, however, indicated a small, marginally significant negative effect (mean difference −0.01 (per 100 patients) CI: −0.01 to 0.00, *p* = 0.053), suggesting that larger clinics might have had slightly lower reach in the weighted model (adjusting rates for clinic size). This effect was not consistently observed, suggesting that clinic size was a minor or negligible factor. ETI analyses did not show a significant effect of post-implementation on reach, reinforcing that time-since-exposure to the implementation strategies did not substantially drive changes in reach. Across models, the most consistent predictors of increased reach of cPRO were time (later study quarters) and cluster effects (higher reach in specific clusters). These findings suggest that changes in cPRO reach may be better attributed to site characteristics and the progression of time rather than the implementation strategies in the post-implementation phase or clinic size. Reach of enrollment in cPRO as an important implementation outcome is supported by cPRO adoption rates across the study period: at the beginning of the study 75% of cPRO completions occurred through the patient portal (MyChart), meaning 25% of cPRO completions occurred via telephone outreach or tablet in the waiting room (i.e., high-burden approaches). By the end of the study period, cPRO was nearly universally completed via the patient portal (98%).

The post-implementation period generally showed a positive effect on the reach of cPRO alert response as measured by proportion of cPRO scores that triggered an alert with dot phrases in response, with significant increases relative to pre-implementation phase levels. In the primary model, the effect on the reach of alerts was positive compared to the pre-implementation period but did not quite reach significance (mean difference = 0.08, CI: −0.01 to 0.18, *p* = 0.10,), with a risk ratio of 1.66 (CI: 0.79 to 3.48). However, study quarters displayed notable temporal effects and when interpreted in conjunction with the implementation indicator, the overall effect of implementation was negative. A sensitivity analysis excluding study quarters supports this observation (post to pre mean difference −0.16, CI: −0.21 to −0.12). Early quarters frequently yielded significant positive estimates, indicating higher reach. In contrast, later quarters often showed significantly declining rates compared to pre-implementation. Cluster effects were significant across most models, with 4 clusters consistently surpassing the reference cluster (Cluster 2) and one cluster showing lower rates of adoption than the reference clinic (primary model mean differences: 0.42 CI: 0.32 to 0.52; 0.45 CI: 0.36 to 0.55; −0.12 CI: −0.23 to −0.01; 0.45 CI: 0.35 to 0.55; 0.52 CI: 0.39 to 0.65). A sensitivity analysis testing the effect of clinic in a linear mixed effects model with Kenwood-Rogers small cluster correction showed the overall effect of clinic in the model was significant (estimate = 0.28, *p* = 0.02). Clinic size consistently had a small, statistically significant, positive impact on reach of clinician alerts, implying that larger clinics achieved slightly higher rates (mean difference = 0.02, CI: 0.02 to 0.02, *p* < 0.001). Effectively, each 100 additional clinic patients was associated with an adoption rate increase of 2%. Exposure quarter effects showed cumulative averaged exposure time estimate was positive and significant gains across exposure periods (time averaged treatment effect (mean difference): 0.35, CI: 0.12 to 0.58), with significant increases in reach starting in exposure quarter 3 and continuing intermittently through later quarters.

## Discussion

Digital symptom management systems for monitoring patient reported outcomes can reduce symptom burden and improve health outcomes, but integration into routine cancer care remains limited. A variety of factors inhibit effective implementation from patient, clinician, and healthcare team and system levels, although few studies have specifically tested and evaluated implementation strategies. Studies that have examined implementation of such interventions have shown that multilevel, multicomponent strategies are required to achieve significant changes in adoption, reach, and other outcomes [12, 14, 16, 17]. This study, one of three hybrid effectiveness-implementation studies using randomized trial designs in the IMPACT Consortium [18], aimed to better understand the strategies that are effective for implementing cancer symptom monitoring and management interventions in routine cancer care centers.

Findings from our cluster randomized stepped-wedge trial did not show a significant effect of system/care team-, clinician-, and patient-level strategies on rates of patient cPRO adoption from pre- to post-implementation periods. However, adoption rates significantly increased over time, and there was significant variation in adoption rates between clusters. Overall, the primary outcome model suggests that time (later study quarters) and cluster are the key drivers of patient adoption of cPRO, but the implementation strategy package itself did not have a significant impact compared to pre-implementation rates accounting for these variables. We hypothesized that effects might be gradual rather than instantaneous; post hoc ETI analyses did not support this hypothesis. Secondary implementation outcome analyses were largely consistent with the primary outcomes in that the implementation strategy package did not meaningfully impact reach rates of cPRO despite an upward trend over time.

Across the three implementation outcomes, clinic size was consistently associated with adoption and reach outcomes, but not always positively related to implementation outcomes. Patient outcomes (adoption of cPRO and reach via patient enrollment in cPRO) varied between clusters with the overall effect showing that the number of patients in the cluster did not significantly affect adoption rates. Clinician outcomes (via adoption of referrals) were consistently positively associated with larger clinic size. This finding may be attributable to greater resource availability (perceived or actual) at larger clinics; for example, the perception among oncologists that palliative care resources are limited has been found to limit referrals [36].

### Contextual considerations

An important consideration about the context of implementation of cPRO in the NM system was that PROs were already being used across the health system, albeit with low patient adoption (about 25% at start of study). Thus, instantaneous effect of the implementation strategy was not possible, limiting our ability to detect effects on reach and adoption of the cPRO symptom monitoring and management program. Implementation scientists could pay better attention to the implications of different scenarios underlying implementation trials where some are a) implementing something new where nothing existed to address the clinical problem previously, b) trying to improve an existing practice that has poor implementation, or c) replacing a practice with a new evidence-based intervention that is thought to be more effective or implementable. The challenge of counteracting behavioral inertia—the tendency to stick to current behaviors and resist change—should not be underestimated, and study design and implementation strategies are best aligned with the context of current practice and the change being sought. This is an area in need of greater attention in implementation research.

The COVID-19 pandemic impacted this trial. While originally scheduled to start March 2020, we delayed initiation. However, when we began later in 2020, care was provided remotely for many, and data capture devices were not allowed into clinics due to concerns for infection control. This had a dampening effect on cPRO adoption across the healthcare system.

### Design considerations

The study design presented challenges to observing an effect of the implementation strategy package. A stepped-wedge trial design assumes that effects of an exposure, in this case the implementation strategies, occur at or around a prespecified point in time. However, this assumption can be difficult to satisfy in implementation trials where some strategies cannot be precisely “turned on” at a given time. In this study, we worked closely with the NM health system to deploy implementation strategies in the package in conjunction with the cross-over point of the stepped-wedge design. Although there were no protocol violations/significant deviations concerning strategy timing, real-world logistics led to some strategies starting just prior (e.g., training clinicians, enlisting leadership and champions), precisely on schedule (e.g., posting posters, turning on reminders to patients and clinicians), or slightly delayed (e.g., monthly newsletters, problem-solving workflow challenges after deployment). Sensitivity analyses that removed one month of data on each side of the crossover did not meaningfully change the results, but questions remain regarding the precision of the timing of the exposure and its relationship to an effect. Thus, stepped-wedge implementation trials might be better suited for strategies that can be deployed precisely at the cross-over point and whose outcomes can be expected to be promptly perceptible.

Our choice of monthly (cross-sectional) rates of adoption and reach across the study period to test an average clinic-level effect of strategies, rather than the average patient- or clinician-level effect, resulted in a small number of datapoints in the analysis. Further, the six-cluster design is small for a stepped-wedge trial [37], which affects statistical power to detect small effects. A parallel cluster randomized trial might have been preferred for testing such a package.

The other two studies in the IMPACT Consortium similarly tested a multilevel package comprising many discrete strategies. The Enhanced, EHR-facilitated Cancer symptom Control trial (E2C2) Research Center [21] deployed 33 discrete strategies in a single, large health system [15] and the Symptom Management Implementation of Patient-Reported Outcomes in Oncology (SIMPRO) Research Center [20], which included six health systems (or sites), used 35 ‘foundational’ strategies across all systems and an additional 64 unique strategies developed and used by the sites beyond the foundational strategies [14]. They further classified these 64 strategies as either ‘universal’, meaning consistently used by multiple sites (*n* = 29), or ‘adaptive’, defined as used only by one of the sites (*n* = 35). Across the studies in the IMPACT Consortium, many strategies targeting multiple levels in the system and addressing contextual factors across determinant domains were necessary to support implementation, but testing them as a package limits the ability to definitively attribute effects, or lack thereof, to any particular discrete strategy.

### Limitations

This study had some limitations. For example, we were limited in our ability to fully assess clinician adoption. Beyond the use of the dot phrase, there was no clear way to assess clinician engagement with cPRO results when the patient didn't trigger an alert (e.g., mild-moderate symptoms) or at the level of reviewing results and/or discussing them with patients during a clinical encounter. The disruptions to the health system related to mitigating the COVID-19 pandemic likely influenced the results, in ways we cannot fully interpret. This study occurred in one health system, which limits generalizability. The study also leveraged existing and well-practiced referral patterns to select specialties (e.g., to the survivorship clinic and Supportive Oncology), but also with limited systematic ability to address referral patterns to other specialties (e.g. rehabilitation services).

## Conclusions

Despite significant effort and investment in supporting adoption and reach of the cPRO cancer symptom monitoring intervention, the results of this stepped-wedge cluster randomized trial did not provide evidence that our multicomponent, multilevel implementation strategy effectively increased adoption or reach of cPRO among patients, though adoption increased over time and clinician referrals also increased.. This study highlights the complexity of evaluating implementation strategies in busy, messy, real-world clinical settings, in which strategies may require prolonged change management, and uptake or compliance may be highly variable across clinicians and practices. Future studies should continue to test strategies to enhance the integration of digital symptom management systems into routine cancer care to improve patient outcomes. They will need to develop designs and methods capable of evaluating the degree of uptake, and isolating the effect, of each discrete strategy (or prespecified strategy bundle). Emerging methodologies in implementation science may enhance our ability to design such real-world studies in the future.

## Supplementary Information

Additional file 1.

## Data Availability

In accordance with Cancer Moonshot grant guidelines, data are to be publicly available once published. An online link is forthcoming, but until that portal is live, please send all requests for data as written proposals to the corresponding author.

## Abbreviations

cPRO: Cancer symptom assessment and management program
EHR: Electronic health record
ETI: Exposure time indicator
IMPACT: Improving the Management of symPtoms during And following Cancer Treatment
LISTS: Longitudinal Implementation Strategies Tracking System
NU IMPACT: Northwestern University IMPACT Study
PROMIS®: Patient Reported Outcomes Measurement Information System®
RCT: Randomized controlled trial
RE-AIM: Reach, Effectiveness, Adoption, Implementation, and Maintenance
